# Predicting Thermal Conductivity of Foamed Concrete via Physics-Informed Neural Networks Coupled with the Maxwell–Eucken Model

**DOI:** 10.3390/ma19204411

**Published:** 2026-10-20

**Authors:** Pengfei Pan, Yakun Zhang, Rui Li, Zheng Wang, Xiaolong Ma

**Affiliations:** 1 Xinhua College of Ningxia University, Yinchuan 750021, China; pengfei_pan@163.com (P.P.); wangzshxc@163.com (Z.W.); 2 Ningxia Center for Research on Earthquake Protection and Disaster Mitigation in Civil Engineering, Yinchuan 750021, China; 3 State Key Laboratory of Fluid Power and Mechatronic Systems, Zhejiang University, Hangzhou 310027, China; ykzhang@zju.edu.cn; 4 School of Mechanical Engineering, Ningxia University, Yinchuan 750021, China

**Keywords:** foamed concrete, thermal conductivity, physics-informed neural networks, Maxwell–Eucken model, predictive modeling, deep learning, energy efficiency

## Abstract

This study investigates a neural network model regularized by the Maxwell–Eucken formulation for predicting the thermal conductivity of foamed concrete. Backed by experimental measurements, the modeling workflow incorporates augmented samples derived from the empirical dataset. A total of 30 experimental records, categorized into five distinct mixture groups, provide the foundational data basis. Bulk density and density-derived porosity serve as model inputs, both reflecting the same underlying variation in material density. Evaluation results demonstrate that the proposed hybrid model achieves a coefficient of determination (R^2^) of 0.7701, substantially outperforming the standalone Maxwell–Eucken model (R^2^ = 0.4546) and the effective medium theory (R^2^ = 0.3041). Although these indicators reflect an improved in-sample fit, they neither isolate the explicit contribution of the physics-informed penalty nor establish the model’s predictive capability for unseen mixtures. The Maxwell–Eucken term functions as an approximate constitutive regularizer, whereas the clustered residuals highlight specific boundary conditions where further model refinement is warranted. Overall, this study validates the feasibility of the hybrid modeling approach within the tested material domain; group-separated validation and comparative benchmarks against purely data-driven models are required to assess its broader generalizability.

## 1. Introduction

Foamed concrete has emerged as a promising construction material, characterized by its distinctive porous structure, lightweight nature, superior thermal insulation, and ease of application [1,2,3]. With growing environmental concerns and stringent energy efficiency regulations worldwide, foamed concrete is becoming increasingly popular in modern construction industries for insulation and structural purposes [4,5,6]. Its notable advantages, including excellent heat insulation, soundproofing, fire resistance, and relatively low density, make it particularly attractive for sustainable building solutions, significantly reducing both energy consumption and environmental footprint [7,8,9]. However, the accurate prediction of its thermal conductivity—a critical parameter influencing thermal performance—remains challenging due to the inherent complexity and heterogeneity of its porous microstructure [10,11,12].

Thermal conductivity significantly impacts the energy efficiency and overall thermal comfort of buildings [13,14,15,16]. Accurate and reliable modeling of thermal conductivity is essential for optimizing material properties, achieving precise energy simulations, and ensuring effective thermal insulation performance in practice [14,17,18]. Traditional methods, including empirical correlations and analytical models, have been extensively employed to predict thermal properties. Among these, the Maxwell–Eucken model [19], a classic analytical framework, has been widely used for its theoretical simplicity and robust physical grounding. This model considers the effective thermal conductivity of heterogeneous two-phase systems, typically comprising a continuous matrix and dispersed inclusions, providing reasonably accurate predictions for materials with relatively simple or uniform microstructures [20,21,22,23].

Despite its popularity, the Maxwell–Eucken model is subject to several intrinsic limitations, notably the assumption of spherical and uniformly dispersed inclusions without interconnections [24]. These assumptions rarely hold true for foamed concrete, whose porous structure often involves complex geometries, irregular pore distributions, and varying levels of pore connectivity. Such structural complexities inevitably induce discrepancies between predicted and experimentally measured thermal conductivities. Furthermore, the material’s thermal performance is sensitive to variations in pore characteristics, moisture content, density, and temperature—factors that traditional analytical models often neglect or oversimplify. Consequently, conventional theoretical models alone cannot reliably capture the detailed thermal behaviors of foamed concrete in realistic applications, prompting a need for more advanced modeling techniques [25,26,27,28].

Recently, machine learning approaches, particularly deep learning, have been introduced to address such complex nonlinear problems due to their powerful approximation capabilities and adaptability to intricate data relationships [29,30]. Deep learning algorithms can effectively capture nonlinear patterns between material characteristics and thermal properties directly from experimental data without explicit physical assumptions. Nevertheless, purely data-driven approaches also possess limitations, such as their dependence on large datasets, susceptibility to overfitting, and lack of interpretability or physical insight, thereby limiting their robustness and applicability in cases of sparse or uncertain data [31].

Physics-informed neural networks (PINNs) incorporate physical equations or model-based penalties into data-driven training [32,33,34]. These approaches combine physical information and data fitting [35]. Such information can regularize learning [36,37], but improved accuracy and generalization must be established through appropriate validation. Although physical consistency motivates this approach [38], the Maxwell–Eucken relation is an approximate constitutive model, not a complete description of the pore-scale heat-transfer problem.

Applications of physics-informed learning to porous building materials motivate investigation of a Maxwell–Eucken-regularized neural network for foamed concrete [39,40]. The physical model supplies a reference trend, while the neural network represents departures from that approximation. Whether this regularization improves prediction over a data-only network remains an empirical question.

In this study, we examine a predictive model combining the Maxwell–Eucken formulation with a neural network using experimentally measured thermal conductivity and density, together with density-derived porosity. Statistical augmentation generates additional modeling samples from those experimental measurements; it does not generate additional independent experimental evidence. This distinction is important when interpreting the sample count and the scope of model validation.

The study explores a hybrid constitutive-regularization framework and visualizes the evolution of network and model parameters. These diagnostics describe the fitted model; they do not independently establish physical mechanisms, parameter identifiability, or resistance to overfitting.

## 2. Methodology

### 2.1. Experimental Data Acquisition

Table 1 reports experimental thermal conductivity, apparent density, density-derived porosity, and compressive strength for five mixture groups, A1–A5. It contains six records per group, giving 30 tabulated experimental records, together with reported group-level values. The modeling workflow also uses augmented samples derived from the experimental data. These derived samples support model training but are not additional independent experimental measurements.

**Figure 1:**
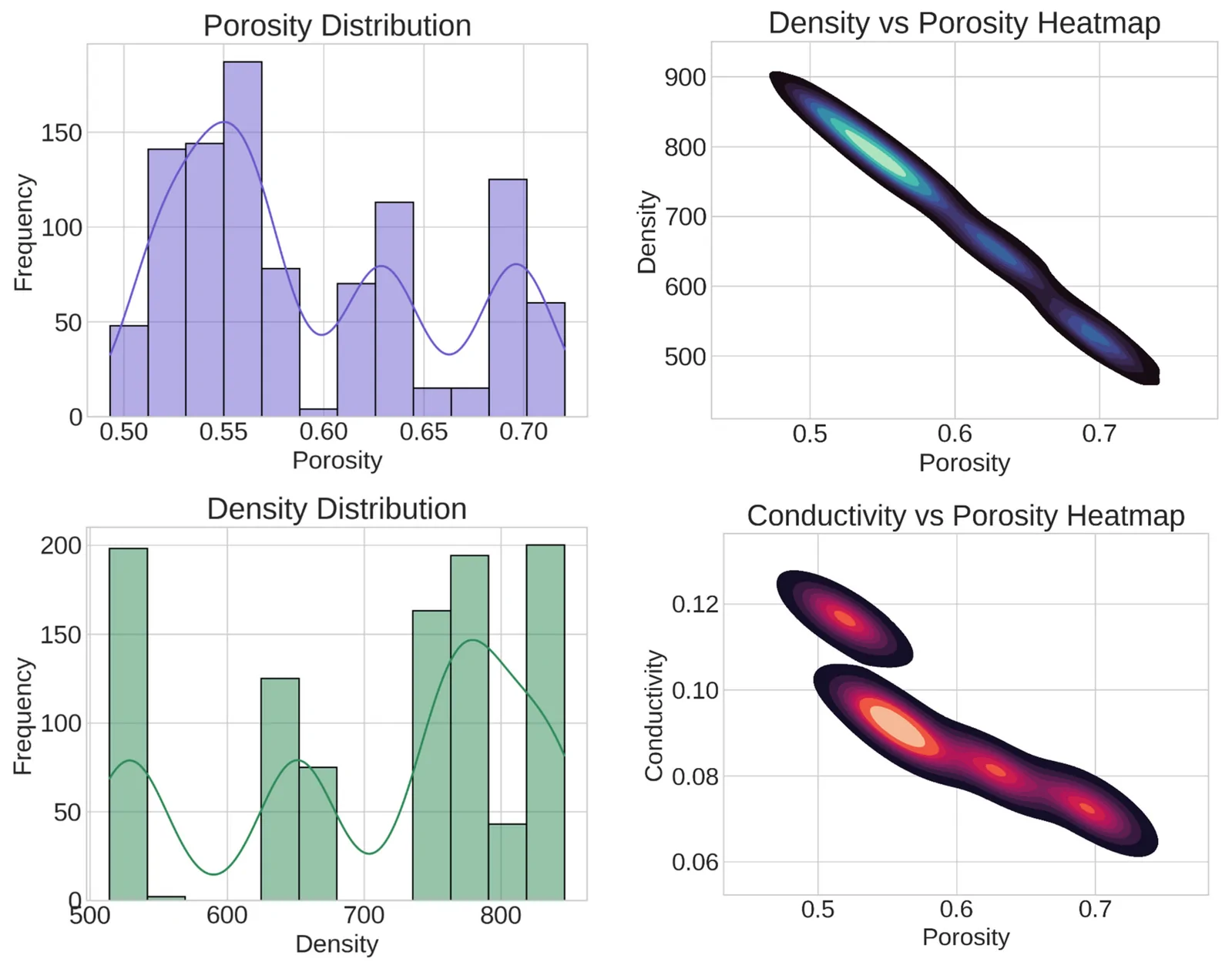

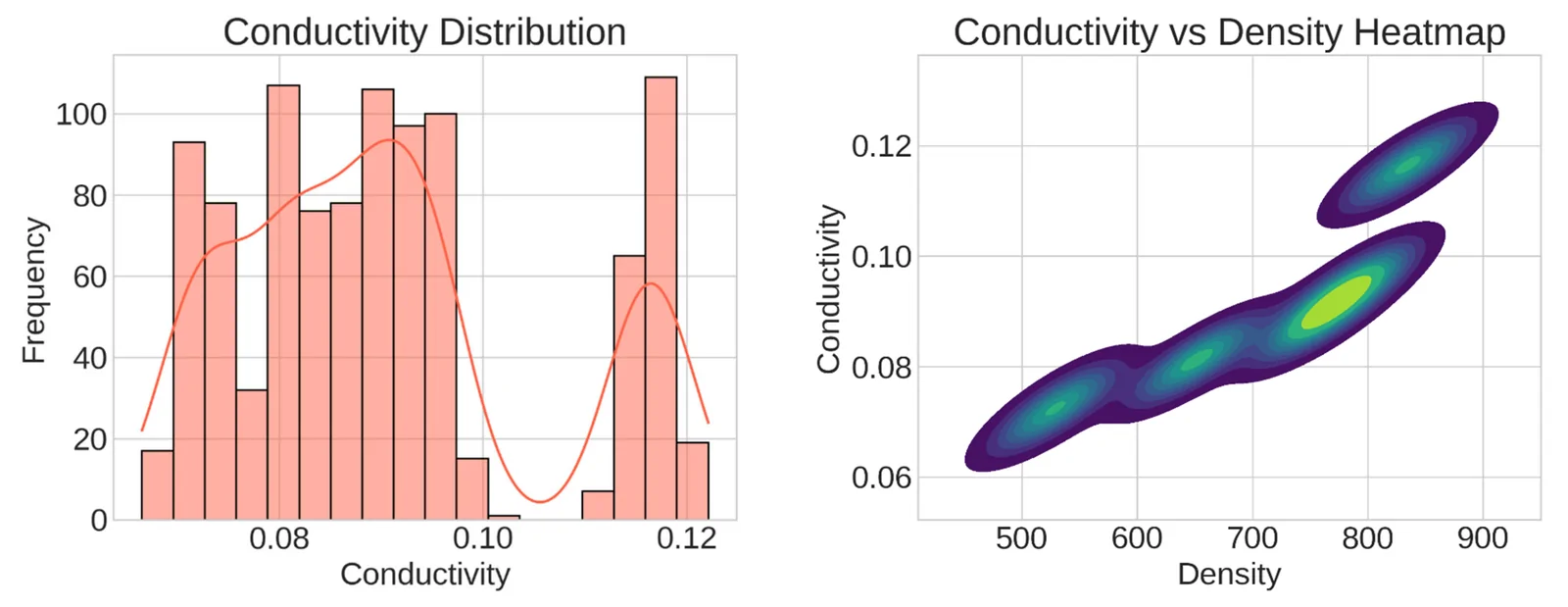
Statistical analysis and correlation of key properties of foamed concrete.

The experimental program comprises five foamed-concrete mixture groups designated A1–A5. Their tabulated densities and porosities differ. The specific procedure is as follows: First, cement and water were added to a mixer in the specified proportions. The mixer was then started and mixing was carried out for 90 s to ensure that the cement and water were thoroughly mixed, thereby producing the base slurry. During this process, a foaming agent solution was prepared, and a foam generator was used to produce uniform and fine foam. The prepared foam was added to the base slurry in a certain volume, and mixing was continued for 30 s to ensure that the foam and slurry were thoroughly mixed and a uniform and consistent mixture was obtained. Figure 1 summarizes the distributions and associations in the modeling dataset, which includes samples derived from the experimental measurements by augmentation. Its multimodal distributions reflect the grouped data structure; augmentation does not expand the number of independently tested mixtures. Because porosity is calculated from density, their negative association is imposed by Equation (2), rather than independently demonstrating a microstructural mechanism.

Thermal conductivity was measured using a thermal conductivity analyzer based on the transient plane source (TPS) method. A foam concrete specimen with a volume of 4 cm^3^ was selected and cut into four layers, each 1 cm thick. During cutting, the cut surfaces of each layer were kept as flat as possible to ensure the accuracy of the test results. The probe of the thermal conductivity analyzer was placed between two adjacent specimen layers, ensuring close contact between the probe and the specimen surfaces, and the assembly was clamped with a fixture to avoid measurement errors caused by poor contact. Parameters such as temperature difference, power, and test duration were adjusted according to the manufacturer’s instructions to ensure that the instrument was in the correct testing state. The thermal conductivity data collected by the sensor in each measurement were recorded, and environmental conditions were kept stable throughout the entire test. The reported group-level conductivity values span 0.0725–0.1166 W·m^−1^·K^−1^, whereas the 30 individual tabulated records span 0.0693–0.1246 W·m^−1^·K^−1^. The lowest and highest reported group-level values correspond to A5 and A1, respectively. Recalculation from the six listed conductivity records gives group means of 0.116600, 0.094867, 0.088617, 0.081083, and 0.072500 W·m^−1^·K^−1^ for A1–A5.

Apparent density was reported as being determined from mass and volume. Porosity was calculated from bulk density and a solid-matrix reference density using Equation (2), rather than measured independently. The individual porosity entries range from approximately 49.08% to 54.76% for A1 and from 67.11% to 71.75% for A5. The final porosity column expresses the corresponding fraction rather than a percentage. Within each property in Table 1, the first column contains the reported group-level value and the next column contains individual records. The group-level values are not uniformly equal to the arithmetic means of those records; for example, the listed densities average 751.53 and 644.10 kg·m^−3^ for A3 and A4, compared with reported group-level values of 758.2 and 650.8 kg·m^−3^.

### 2.2. Maxwell–Eucken Thermal Conductivity Model

The accurate prediction of thermal conductivity in heterogeneous materials such as foamed concrete often requires utilizing physically grounded analytical models. Among these, the Maxwell–Eucken model stands out as one of the classical approaches due to its robust theoretical foundations and practical effectiveness in representing two-phase composite systems. Originally developed by Maxwell for electric conductivity and later adapted by Eucken for thermal conduction problems, the Maxwell–Eucken model assumes a simplified structure wherein spherical inclusions (dispersed phase) are randomly embedded within a continuous matrix phase. This assumption significantly simplifies the complex microstructural interactions inherent in heterogeneous materials, allowing for the derivation of analytical expressions to approximate effective thermal properties.

Mathematically, the Maxwell–Eucken effective thermal conductivity (*k_e_*) for a two-phase heterogeneous material can be expressed as follows:

(1) $$k_{e}=k_{s}\frac{k_{f}+2k_{s}-2\varepsilon (k_{s}-k_{f})}{k_{f}+2k_{s}+\varepsilon (k_{s}-k_{f})}$$

In Equation (1), the effective conductivity of the composite, the solid-phase conductivity, and the dispersed-phase conductivity are denoted by $k_{e}$, $k_{s}$, and $k_{f}$, respectively. Each has units of W·m^−1^·K^−1^. The air-phase conductivity is approximated as 0.026 W·m^−1^·K^−1^; this is a model input and does not specify the experimental test temperature. The dimensionless porosity ε is the void volume fraction and is calculated from measured bulk density ρ and solid-matrix reference density $\rho_{s}$ as follows:

(2) $$\varepsilon =1-\frac{\rho }{\rho_{s}}$$

For a fixed solid-matrix reference density, Equation (2) makes porosity an affine transformation of bulk density. The two variables contain the same one-dimensional information, so their correlation is not independent evidence of pore structure and separate feature-importance claims are not justified. Density-only, porosity-only, and joint-input models must be compared under the same group-separated validation to assess any numerical effect of this redundant representation.

### 2.3. Physics-Informed Neural Networks (PINN) Framework

The network is trained using a data-fitting term and a penalty based on the Maxwell–Eucken relation. Here, the term physics-informed refers to constitutive regularization. Because the relation assumes an idealized two-phase geometry, agreement with it is not equivalent to satisfying the full heat-transfer problem in a connected pore network.

**Figure 2:**
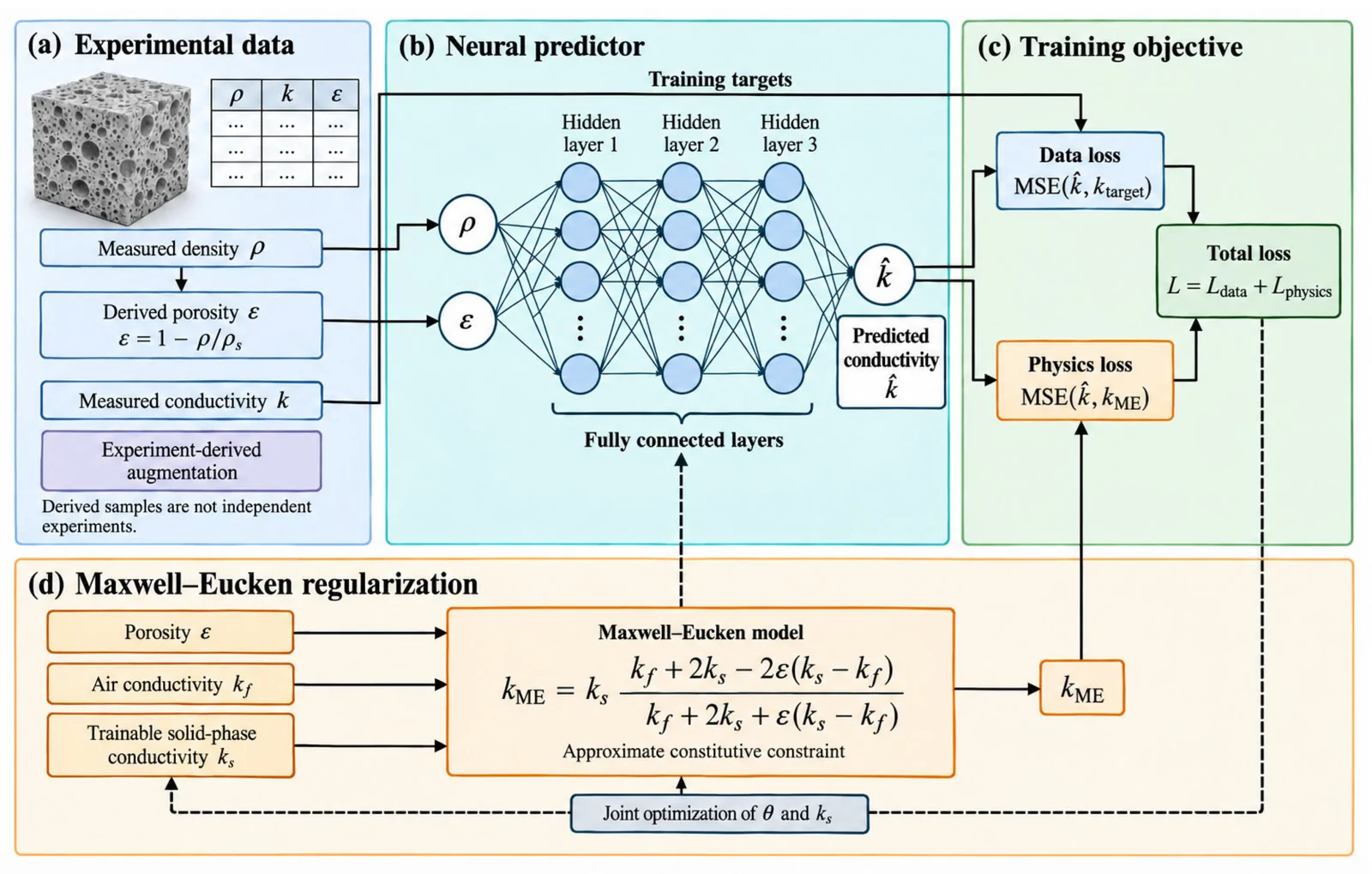
The proposed PINN framework.

The network maps porosity and density to thermal conductivity through fully connected hidden layers and an output layer, as illustrated in Figure 2. The activation diagnostics in the Section 3 are labeled as three hidden layers. Layer count, widths, and activation functions must be consistent with the implementation used to generate the predictions.

A physics-based loss term penalizes disagreement between the neural-network prediction and the Maxwell–Eucken effective conductivity. This is a soft penalty, so minimizing it does not guarantee exact agreement with the physical model or with the true pore-scale physics:

(3) $$k_{e,phy}(\varepsilon ,k_{s})=k_{s}\frac{k_{f}+2k_{s}-2\varepsilon (k_{s}-k_{f})}{k_{f}+2k_{s}+\varepsilon (k_{s}-k_{f})}$$

The PINN’s overall loss function combines two essential components: the data-driven loss (empirical observations) and the physics-based loss (derived from the Maxwell–Eucken analytical model):

(4) $$L(\theta ,k_{s})=\underset{\text{Data-driven}\;\mathrm{Loss}}{\underbrace{\frac{1}{N}\sum_{i=1}^{N}\;(k_{pred,i}-k_{true,i}{)}^{2}}}+\underset{\text{Physics-based}\;\mathrm{Loss}}{\underbrace{\frac{1}{N}\sum_{i=1}^{N}\;(k_{pred,i}-k_{e,phy,i}{)}^{2}}}$$

Here, *N* is the total number of training samples, $k_{true,i}$ denotes experimentally measured thermal conductivity, and $k_{e,phy,i}$ represents predictions derived from the Maxwell–Eucken model using optimized parameters.

Equation (4), as written, adds the two mean-squared loss terms with equal coefficients. Equal nominal weights do not imply equal influence on optimization, because the magnitudes and gradients of the terms may differ. The equation does not establish the weighting actually used in the implementation.

The solid-phase conductivity parameter $k_{s}$ is optimized together with the network parameters. In this setting it is an effective fitted parameter that may compensate for pore-geometry simplifications, moisture effects, or other unmodeled influences. It should not be identified with an independently measured intrinsic solid-phase conductivity without additional evidence. Equation (5) states formal stationarity conditions; finite optimization does not establish that they are exactly satisfied:

(5) $$\frac{\partial L}{\partial k_{s}}=0,\frac{\partial L}{\partial \theta }=0$$

Optimization seeks to reduce the combined loss. Convergence of that objective alone does not establish a unique physical parameter, an accurate prediction for a new mixture, or the absence of overfitting.

### 2.4. Model Training and Validation Procedures

The reported workflow uses an 80:20 training–validation split. The experimental origin of the source data and the independence of the validation subsets are separate considerations. When experimental records are augmented, a source record and its derived samples must remain in the same subset to avoid leakage. A record-wise split may also place the same mixture in both subsets, so its results should be interpreted as within-dataset performance rather than prediction for an unseen mixture. Leave-one-mixture-out evaluation provides a suitable test of the latter. Augmentation, scaling, parameter fitting, and model selection must be confined to the training data. RMSE, MAE [41], and the coefficient of determination should be evaluated on common held-out original measurements for every model:

(6) $$RMSE=\sqrt{\frac{1}{n}\sum_{i=1}^{n}\;(y_{i}-{\hat{y}}_{i}{)}^{2}},MAE=\frac{1}{n}\sum_{i=1}^{n}\;|y_{i}-{\hat{y}}_{i}|,R^{2}=1-\frac{\sum_{i=1}^{n}\;(y_{i}-{\hat{y}}_{i}{)}^{2}}{\sum_{i=1}^{n}\;(y_{i}-\bar{y}{)}^{2}}$$

## 3. Results and Discussion

### 3.1. PINN Training Performance and Prediction Accuracy

**Figure 3:**
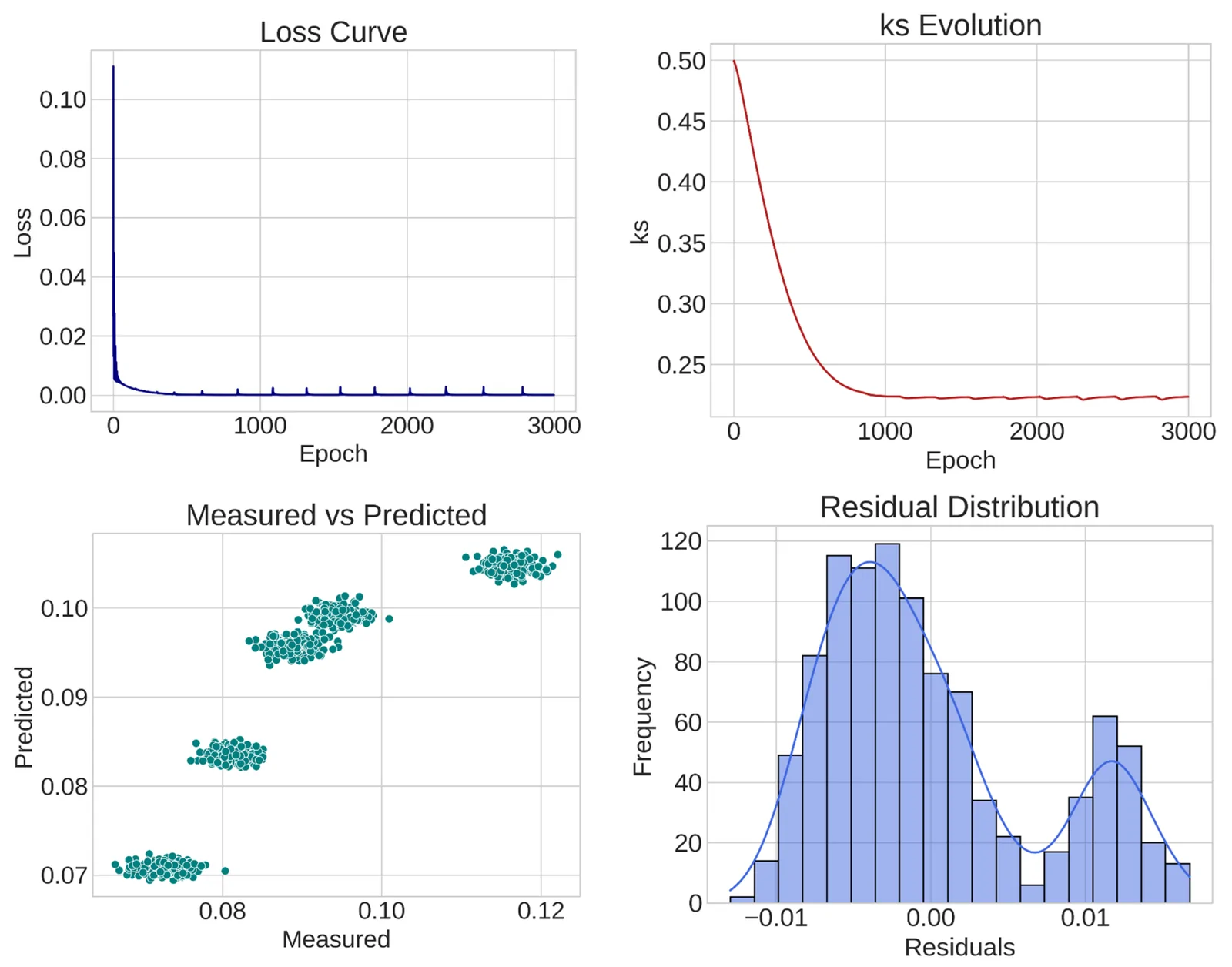
Training performance and prediction accuracy.

Figure 3 shows the reported loss history, evolution of the fitted solid-phase parameter, measured-versus-predicted values, and residual distribution. The decreasing loss and leveling parameter trajectory indicate numerical stabilization in that run, but do not establish parameter identifiability or generalization. An approximately centered pooled residual histogram does not demonstrate normality or exclude mixture-specific bias. Its interpretation must be considered together with the layer-wise residual analyses discussed below.

### 3.2. Analysis of Network Internal Parameters

**Figure 4:**
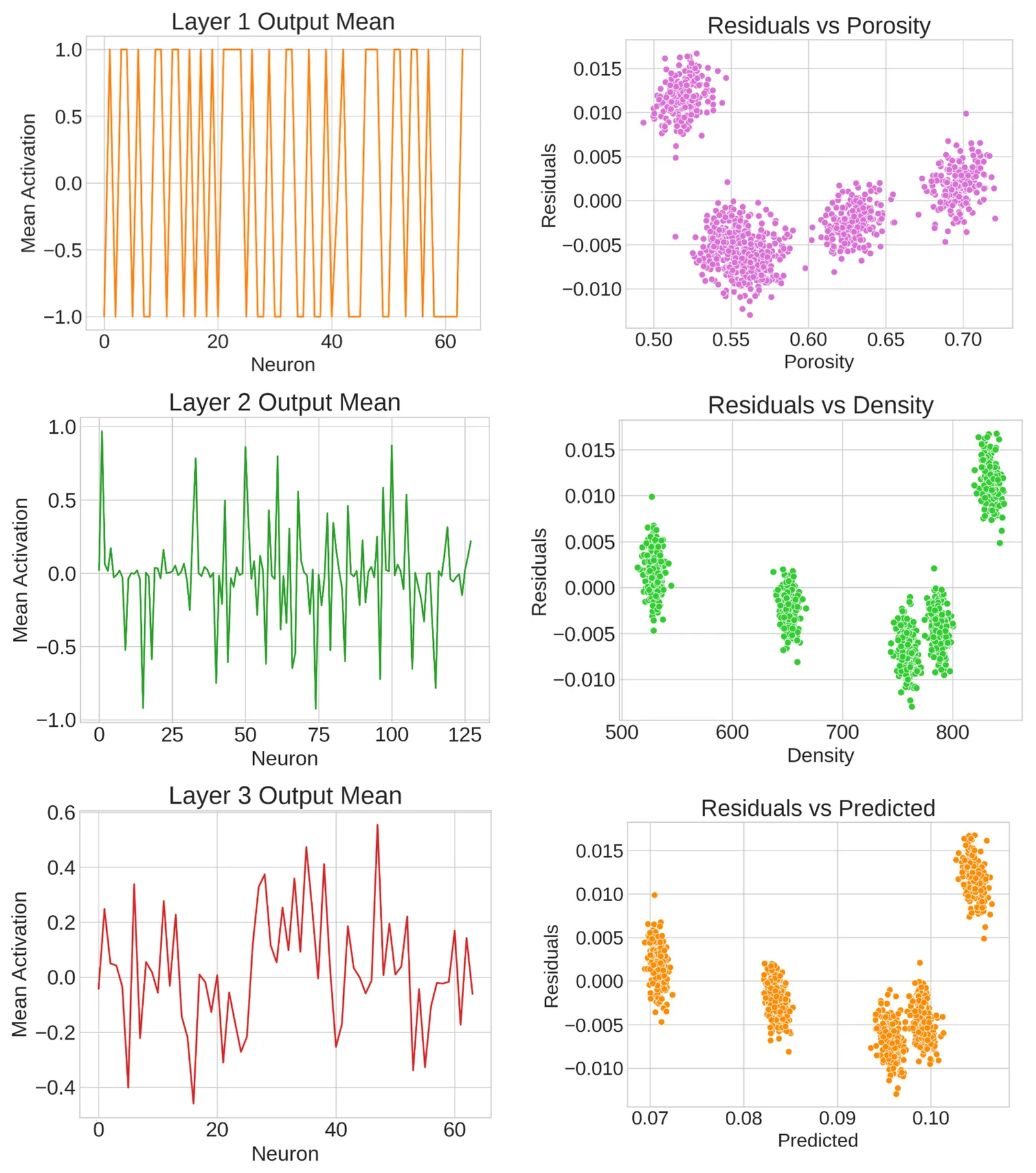
Analysis of PINN internal activations and residual relationships.

Figure 4 shows layer-wise activation summaries and residuals against porosity, density, and predicted conductivity. Differences in activation levels are descriptive properties of this fitted network; they do not establish distinct physical mechanisms. The residual clusters indicate that prediction errors vary across material groups or input ranges. Because density and porosity are deterministically related, the two input-residual plots are alternative views of the same dependency.

**Figure 5:**
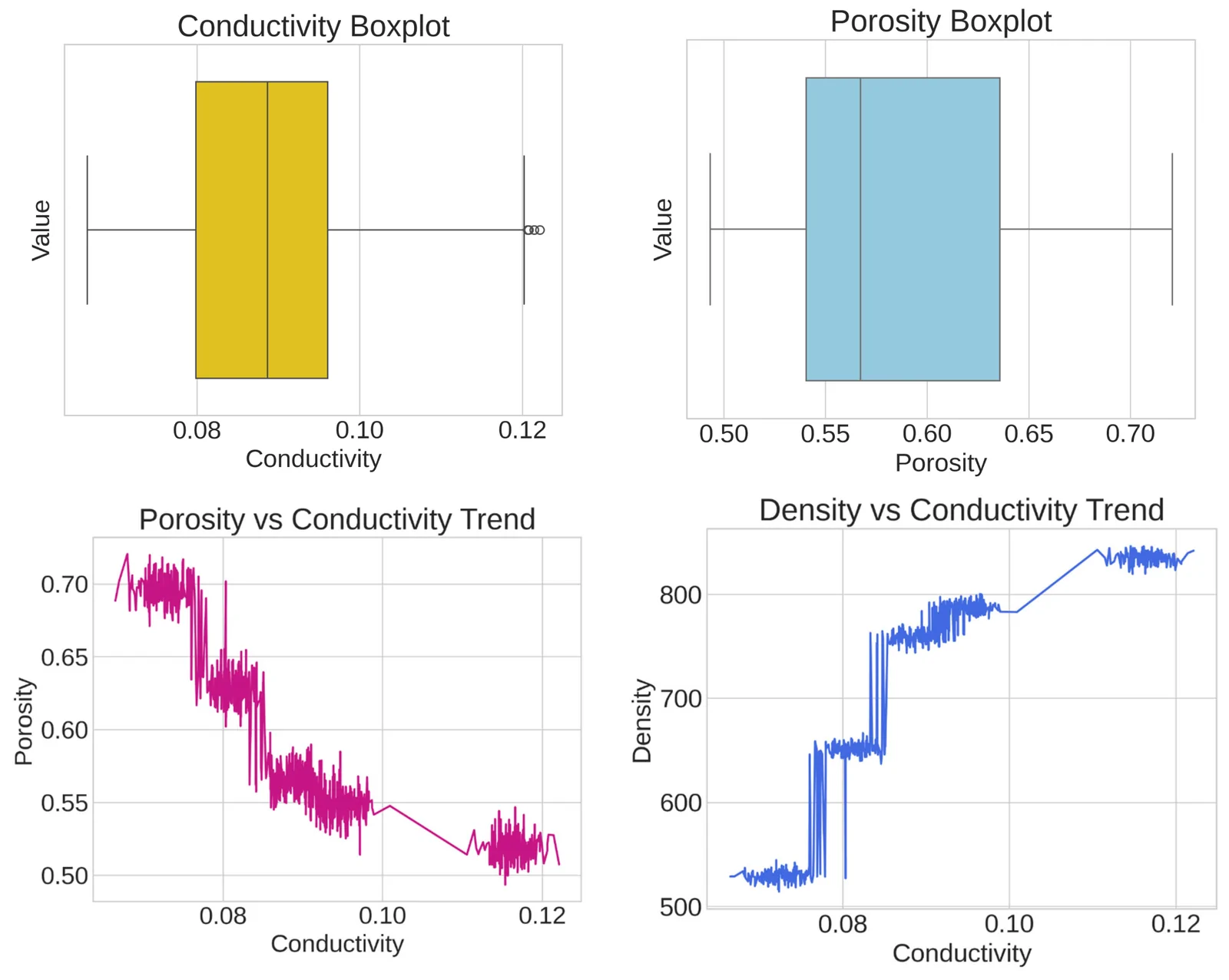
Predicted thermal conductivity trends with porosity and density.

Figure 5 shows decreasing predicted conductivity with increasing porosity and increasing predicted conductivity with density over the plotted data range. These trends are compatible with the two-phase model. However, because porosity is derived from density, the panels do not demonstrate two independent physical effects or establish predictive validity outside the studied mixtures.

### 3.3. Activation Diagnostics and Residual Structure

**Figure 6:**
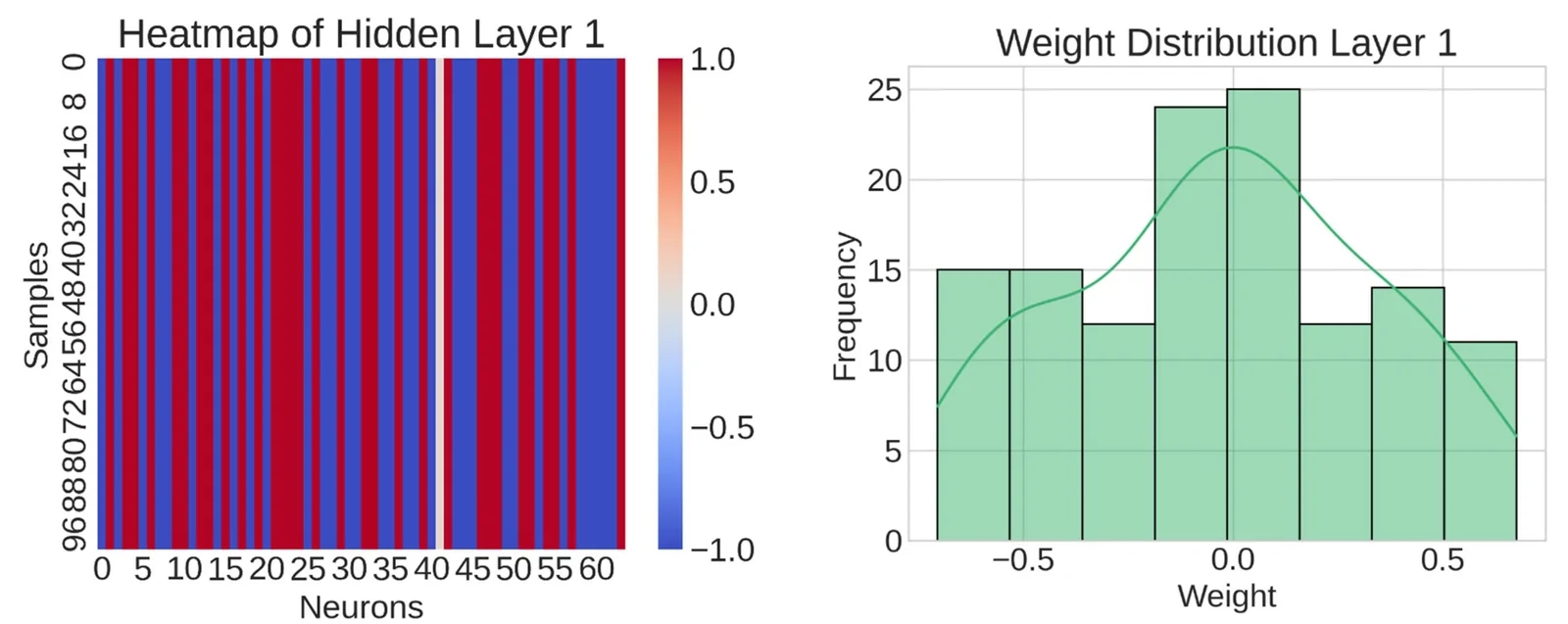

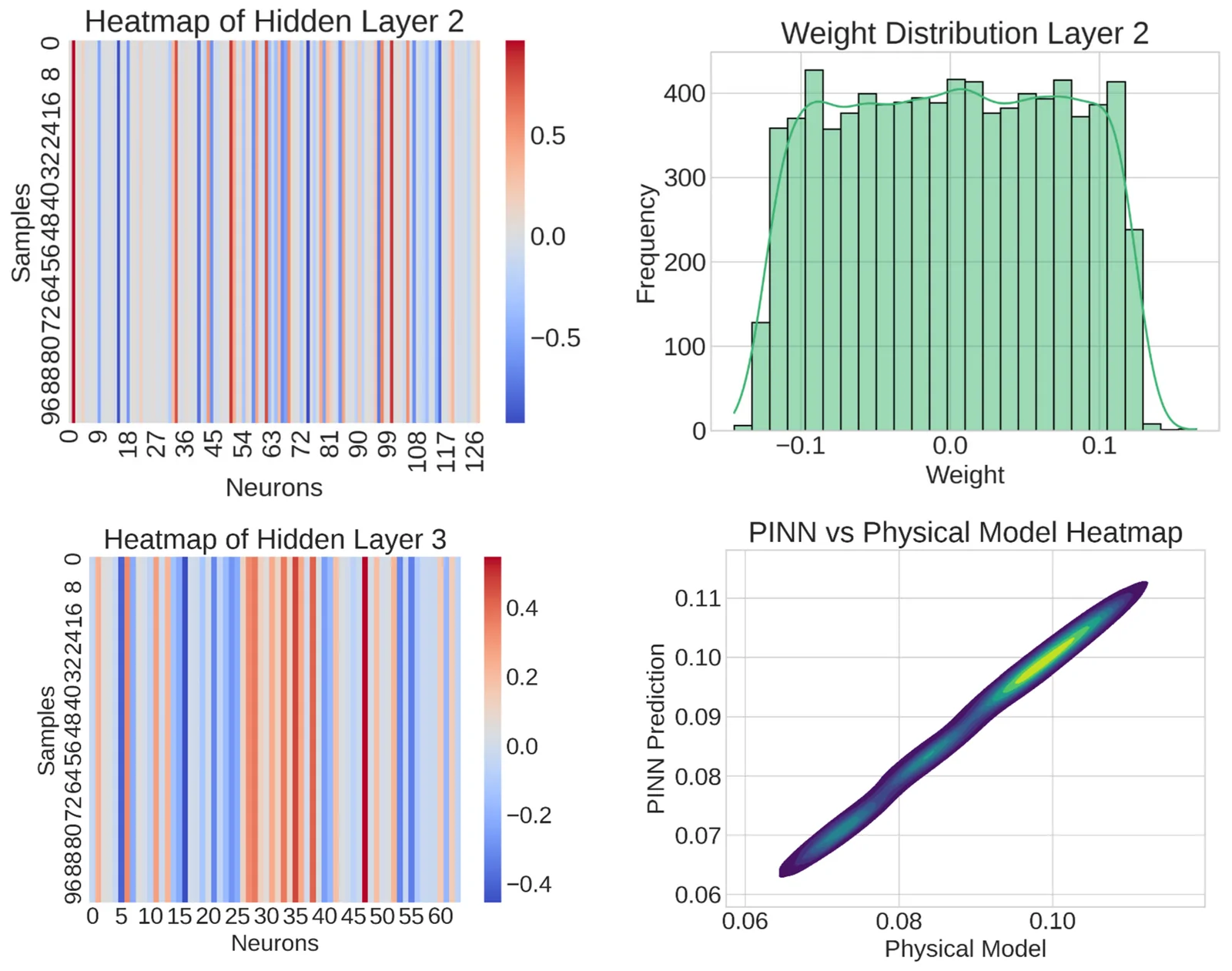
Internal Activation Patterns, Weight Distributions.

Figure 6 displays activation heatmaps, weight distributions, and agreement between network and Maxwell–Eucken predictions. Activation saturation or a near-normal weight distribution does not demonstrate successful physical feature extraction or exclude overfitting. Likewise, agreement with the Maxwell–Eucken relation is partly encouraged by the training loss and is not an independent validation of that relation. A matched network without the physics term and group-separated prediction errors are needed to determine the contribution of constitutive regularization.

**Figure 7:**
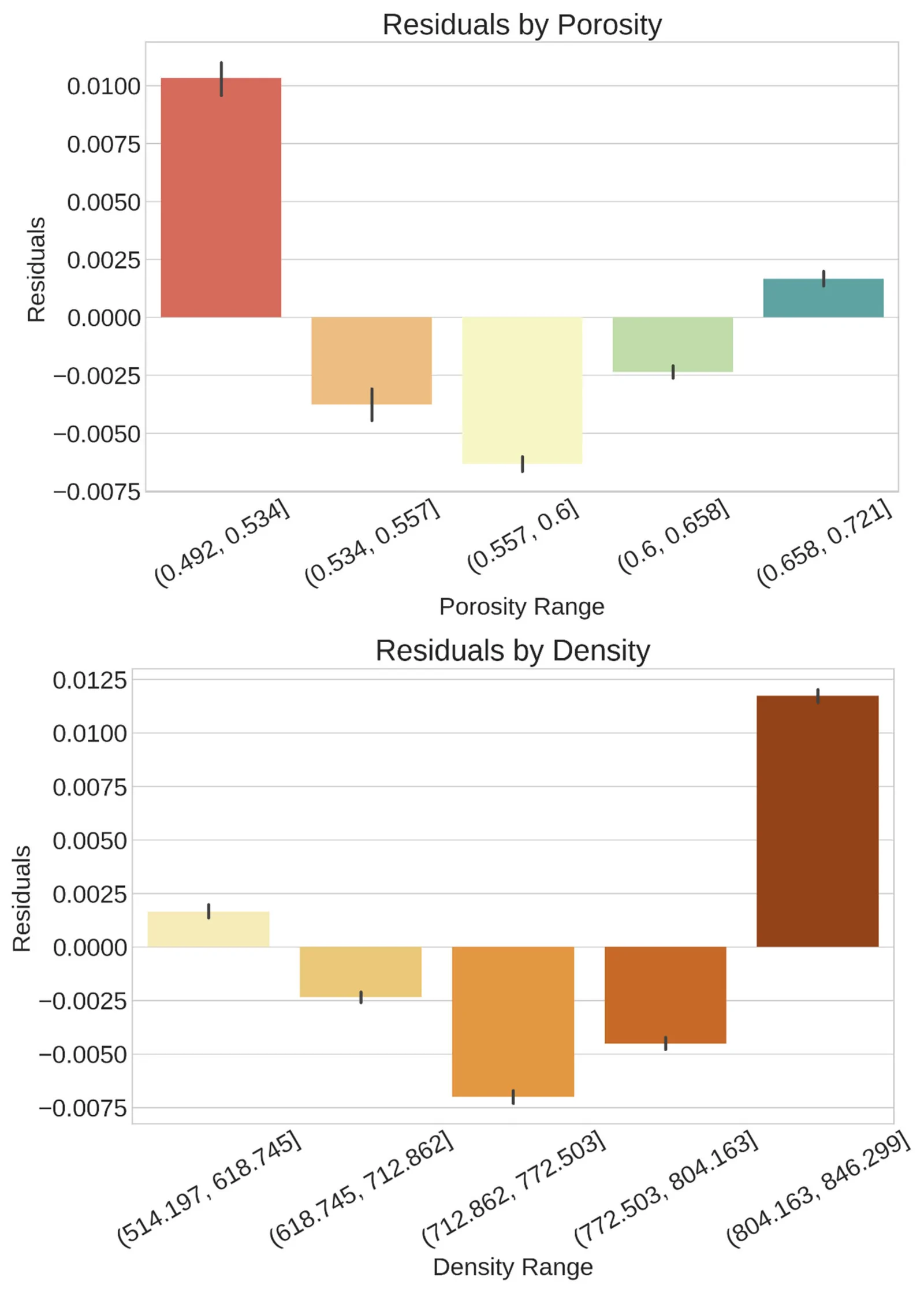

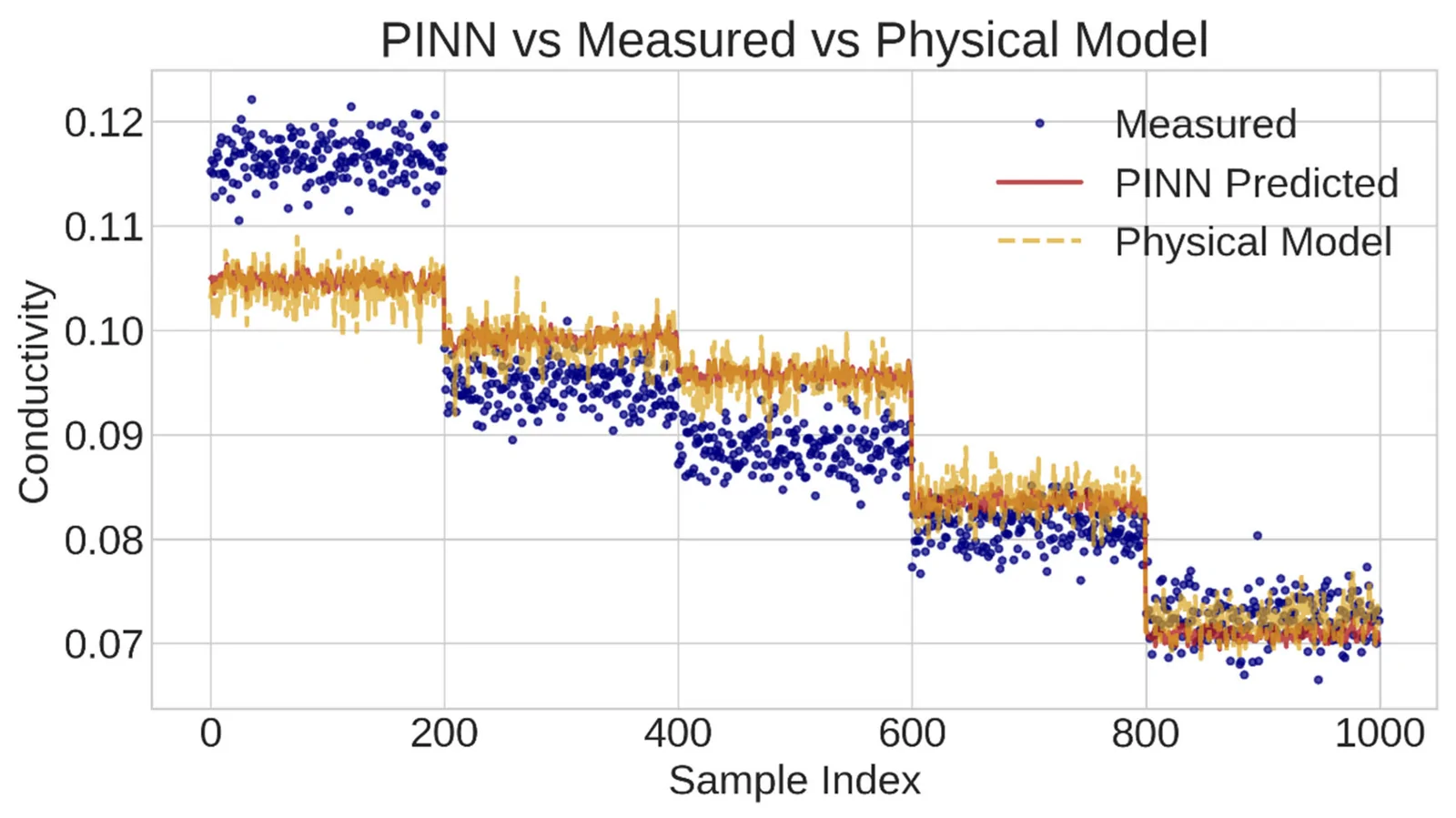
Residual analysis and comparative evaluation of the PINN predictions.

Figure 7 displays residuals binned by porosity and density and compares measured, Maxwell–Eucken, and network-predicted conductivity. Under a predicted-minus-measured convention, positive residuals indicate overprediction and negative residuals indicate underprediction. The sign convention of the existing plots must be verified against the point-wise prediction records before assigning those interpretations. The binned patterns indicate systematic error across portions of the input range, even if pooled residuals appear approximately centered. Mixture-specific signed mean error, MAE, RMSE, and observation counts on held-out original records are needed to quantify this bias. The plotted agreement does not by itself establish broad robustness or physical validity.

### 3.4. Comparative Evaluation with Traditional Models

The reported comparison includes Maxwell–Eucken, Parallel, Series, Krischer, and effective medium theory (EMT) predictions. These models embody different idealizations of two-phase heat transfer. A fair predictive comparison requires identical held-out original observations and calibration of every adjustable parameter using training data only. Without a matched data-only network and a simple density-based regression under that same evaluation, the contribution of the physics penalty cannot be isolated.

The Parallel model [42], known for providing an upper-bound prediction of effective thermal conductivity, assumes that each phase within the composite is arranged parallel to the direction of heat flow. Consequently, this approach simplifies the analysis by considering that each phase independently contributes to the overall thermal conductivity based on its volumetric fraction. Mathematically, the effective thermal conductivity (*k_e_*) predicted by the Parallel model can be expressed as

(7) $$k_{e,parallel}=\varepsilon k_{f}+(1-\varepsilon )k_{s}$$

Conversely, the Series model [43] provides a lower-bound estimation, premised on the assumption that heat must sequentially traverse each distinct phase arranged in series. This setup yields conservative predictions for effective thermal conductivity, mathematically formulated as

(8) $$k_{e,series}=\frac{1}{\frac{\varepsilon }{k_{f}}+\frac{1-\varepsilon }{k_{s}}}$$

This model often underestimates thermal conductivity in porous materials because it overemphasizes the resistance to heat flow induced by low-conductivity phases like air, neglecting alternative pathways and interconnections that exist in real porous structures.

The empirical Krischer model [44] attempts to address some of these limitations by combining geometric averaging with volumetric weighting, formulated as follows:

(9) $$k_{e,Krischer}=k_{s}^{\left(1-\varepsilon \right)}\cdot k_{f}^{\varepsilon }$$

This model, though straightforward and easy to apply, relies heavily on empirical relationships and lacks detailed consideration of structural complexities or interaction effects between phases.

The Effective Medium Theory (EMT) model [45], by contrast, assumes a random distribution of phases without a clearly defined dominant continuous matrix, implicitly accounting for mutual phase interactions. The EMT effective thermal conductivity is implicitly described by the following relation:

(10) $$\varepsilon \frac{k_{f}-k_{e,EMT}}{k_{f}+2k_{e,EMT}}+(1-\varepsilon )\frac{k_{s}-k_{e,EMT}}{k_{s}+2k_{e,EMT}}=0$$

Solving this implicit equation numerically provides a prediction of thermal conductivity, effectively capturing more realistic scenarios in materials with complex structural heterogeneity. Nevertheless, EMT still neglects explicit connectivity or detailed microstructural morphology, resulting in moderate accuracy.

Pore size, connectivity, and the proportions of open and closed pores are not resolved by a density-derived total porosity. Specimens with the same total porosity can therefore have different heat-flow paths and moisture access. Water occupying accessible pores changes the phase composition and limits the applicability of a solid–air approximation; moisture is a recognized influence on concrete conductivity [22]. These mechanisms are plausible explanations for deviations from Maxwell–Eucken predictions, not mechanisms identified by the present dataset. The model inputs and Table 1 cannot separate pore-size, connectivity, and moisture effects, so extrapolation across these conditions is not supported.

**Figure 8:**
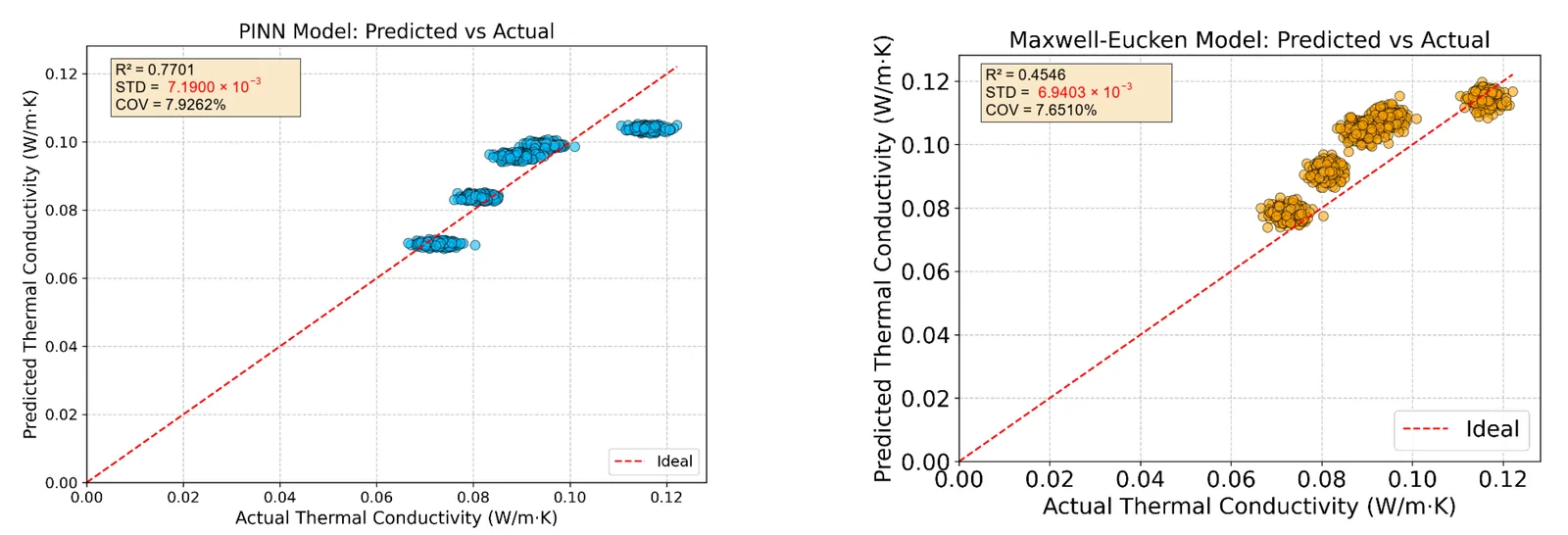

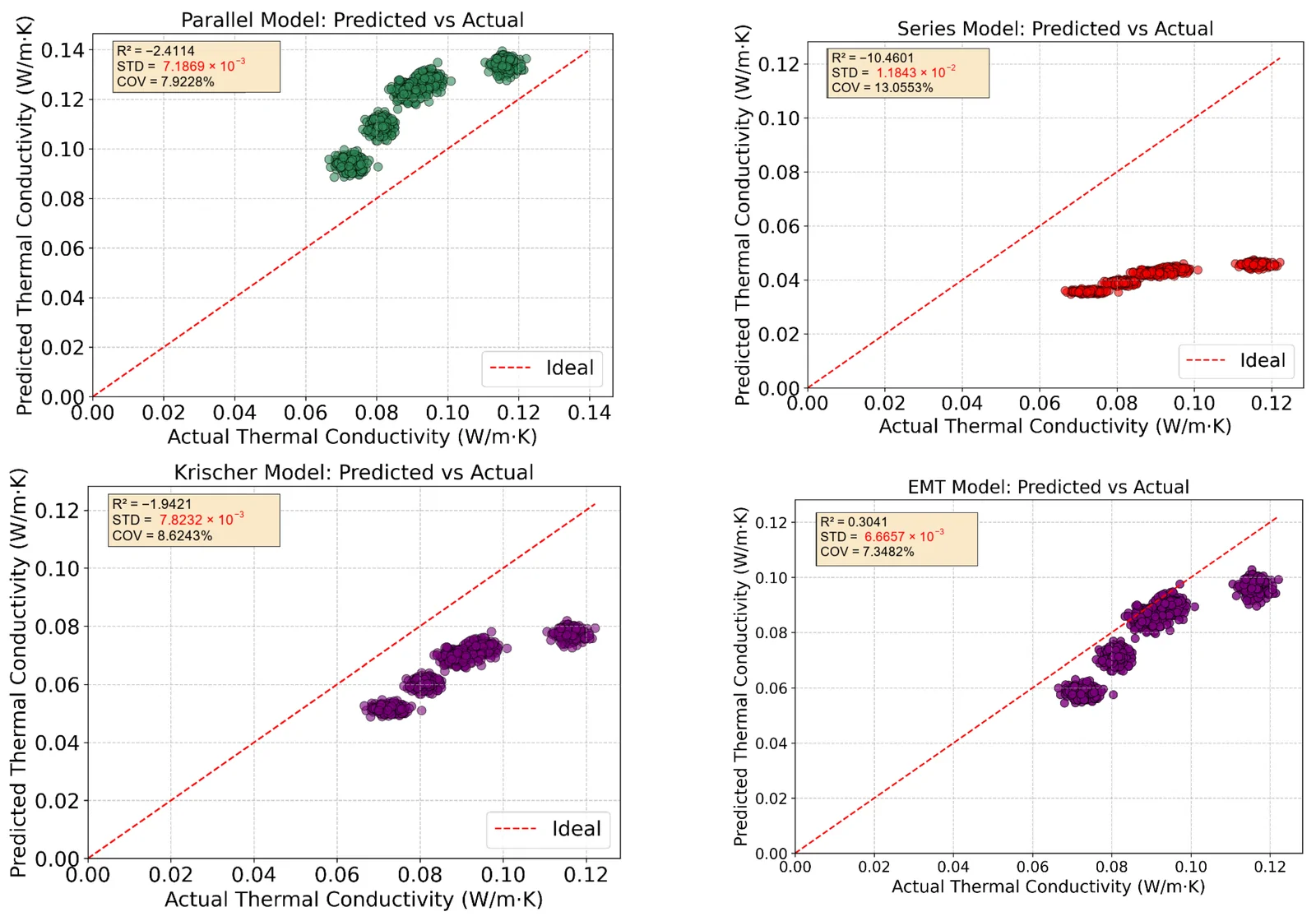
Prediction performance. Colors: cyan, PINN; orange, Maxwell–Eucken; green, Parallel; red, Series; purple, Krischer/EMT. Red dashed lines: Ideal.

Figure 8 reports coefficients of determination of 0.7701 for the hybrid model, 0.4546 for Maxwell–Eucken, 0.3041 for EMT, −2.4114 for Parallel, −10.4601 for Series, and −1.9421 for Krischer. These are the values from the reported comparison, not results from a newly established leave-one-mixture-out test. Their ranking cannot demonstrate a benefit specifically attributable to the physics loss without matched baselines, a common held-out sample set, and training-only parameter calibration. The plotted STD and COV do not specify whether their underlying quantity is the predictions or prediction errors; they therefore cannot establish prediction stability. RMSE and MAE values are also needed on that same held-out set before quantitative conclusions about relative error can be drawn.

## 4. Conclusions

This study examines a Maxwell–Eucken-regularized neural network using the five foamed-concrete mixture groups in Table 1. The reported results illustrate the behavior of the fitted framework, but the present evidence does not establish generalization to new mixtures or isolate the benefit of the physics term. The following conclusions are supported within this limited scope:

(1) The reported hybrid-model coefficient of determination is 0.7701. Because group-independent validation and matched data-only baselines have not been established, this value should not be interpreted as evidence that constitutive regularization improves out-of-mixture prediction.
(2) Density-derived porosity is not an independent input descriptor. The observed conductivity trends therefore cannot be used to attribute separate predictive contributions to density and porosity. Clustered and binned residuals indicate systematic errors that are not ruled out by a centered pooled histogram.
(3) The Maxwell–Eucken relation provides a soft, approximate constitutive constraint. Its optimized solid-phase parameter is an effective calibration quantity rather than a validated intrinsic material property. Group-separated reanalysis, complete experimental documentation, and independent pore and moisture characterization are required before broader physical or predictive claims can be made.

## Figures and Tables

**Table 1:** Experimental results.

Sample	Compression (MPa)		Thermal Conductivity (W·m^−1^·K^−1^)		Apparent Density (kg·m^−3^)		Porosity		
A1	1.736	1.480	0.1166	0.1050	834.1	785.3	51.95%	54.76%	0.547638249
		1.697		0.1169		810.7		53.30%	0.533006912
		1.886		0.1206		880.9		49.26%	0.492569124
		1.824		0.1205		845.2		51.31%	0.513133641
		1.553		0.1120		798.5		54.00%	0.540034562
		1.916		0.1246		884.0		49.08%	0.49078341
A2	1.407	1.258	0.0947	0.0907	787.4	731.6	54.64%	57.86%	0.578571429
		1.448		0.0959		798.1		54.03%	0.540264977
		1.367		0.0928		791.0		54.44%	0.544354839
		1.544		0.0998		831.5		52.10%	0.521025346
		1.522		0.0985		808.7		53.42%	0.534158986
		1.303		0.0915		763.4		56.03%	0.560253456
A3	1.197	1.148	0.0886	0.0877	758.2	700.8	56.71%	59.63%	0.596313364
		1.110		0.0880		715.4		58.79%	0.587903226
		1.098		0.0814		735.6		57.63%	0.576267281
		1.301		0.0936		790.1		54.49%	0.544873272
		1.209		0.0891		765.3		55.92%	0.559158986
		1.316		0.0919		802.0		53.80%	0.538018433
A4	0.878	0.790	0.0811	0.0752	650.8	600.5	62.90%	65.41%	0.654089862
		0.963		0.0876		682.5		60.69%	0.606854839
		0.820		0.0796		615.3		64.56%	0.645564516
		0.935		0.0839		675.4		61.09%	0.6109447
		0.850		0.0778		635.7		63.38%	0.633813364
		0.910		0.0824		655.2		62.26%	0.622580645
A5	0.652	0.586	0.0725	0.0693	528.9	490.5	69.53%	71.75%	0.717453917
		0.720		0.0757		571.0		67.11%	0.671082949
		0.615		0.0705		505.3		70.89%	0.708928571
		0.669		0.0732		550.1		68.31%	0.68312212
		0.633		0.0719		518.2		70.15%	0.701497696
		0.691		0.0744		538.4		68.99%	0.689861751

## Data Availability

The experimental results are included in the article. Selected experimental data will be released on GitHub after publication. Further inquiries can be directed to the corresponding authors.
